# Prognostic significance of preoperative serum triglycerides and high-density lipoproteins cholesterol in patients with non-small cell lung cancer: a retrospective study

**DOI:** 10.1186/s12944-021-01492-y

**Published:** 2021-10-02

**Authors:** Cong Ma, Xiaoyan Wang, Jingjing Guo, Ping Liu

**Affiliations:** 1 Department of Surgery, Liyuan Hospital, Tongji Medical College, Huazhong University of Science and Technology, Wuhan, 430077 Hubei China; 2 Jiashan Maternal and Child Health Hospital, Jiaxing, Zhejiang, 314100 China; 3 Department of Hematology, Union Hospital, Tongji Medical College, Huazhong University of Science and Technology, Wuhan, 430022 Hubei China

**Keywords:** Non-small cell lung cancer, Triglycerides, High-density lipoprotein cholesterol, Prognosis

## Abstract

**Background:**

Abnormalities in serum lipids and lipoproteins have been documented to link to the risk of cancers in recent years, but its prognostic value for cancer is not known. This study retrospectively evaluated the significance of preoperative serum lipids and lipoproteins for NSCLC’s prognosis.

**Methods:**

A retrospective review was implemented of 551 patients succumbed to NSCLC. A ROC curve was utilized to determine the best cut-off value and area under the ROC curve. Kaplan-Meier and a Cox proportional hazards model were utilized to perform survival analysis.

**Results:**

With a median follow-up of 42 months, the NSCLC patients in the high TG (> 1.21 mmol/L) and low HDL-C (≤ 1.26 mmol/L) two groups exhibited shorter OS and DFS. In multivariable analysis, preoperative HDL-C and TG can work as independent prognosis factors for OS (*P*<0.001 for both) and DFS (*P*<0.05 for both) in patients succumbed to NSCLC.

**Conclusion:**

Abnormalities of serum lipids and lipoproteins metabolism linked to the survival outcomes of NSCLC. Preoperative serum HDL-C and TG may be promising biomarkers to predict the NSCLC patients’ prognosis.

## Introduction

Lung cancer remains the most prevalent malignancy worldwide, with almost one-quarter of deaths of cancer patients result from lung cancer [1]. NSCLC is comprised of around 85% of all the lung cancer cases [2]. Though surgical resection is the only potentially cure treatment for NSCLC at present, the average five-year survival rate of all stages remains below 23% [3]. The majority (56%) of NSCLC patients with stages I-II undergo surgery, whereas only 10% of patients at the stage III NSCLC can undergo surgery [4]. In contrast, the most (62%) of NSCLC patients with stages III-IV undergo treatment with chemotherapy, immunotherapy and molecular target therapy [5]. However, the average five-year survival rate of the stage IV NSCLC (4%) is lower than that for stage I (57%) [4, 6]. Therefore, finding accurate, effective and inexpensive pre-treatment biomarkers for prognosis evaluation of NSCLC can be of great clinical value.

Abnormalities in serum lipids and lipoproteins are important causes of cardiovascular disease and putatively associated with cancer [7–9]. Triglycerides (TG) are a component of blood lipids and an independent source of fatty acid oxidation, meanwhile have been associated with cell proliferation and tumor growth, and therefore have carcinogenic potential [10, 11]. Some studies have shown that TG levels positively correlate with the risk of thyroid, rectal, and renal cancer, but negatively with the risk of prostate cancer [12, 13]. Cholesterol is another component of lipids, which can regulate cell membrane stability and participate in lipid raft formation [14]. High-density lipoprotein cholesterol (HDL-C) can mediate reverse transport of cholesterol from cell to liver, and may have an anti-tumor effect because of its anti-oxidation and anti-inflammation properties [15]. The association between high HDL-C and low overall cancer incidence has been confirmed in some epidemiological studies [16, 17].

Nevertheless, only a few studies have indicated an association between serum lipids and lipoproteins and cancer prognosis, and the cancers are site-specific such as breast and colorectal [18, 19]. There are rarely retrospective studies concerning TG, HDL-C, or other lipids parameters and cancer prognosis in NSCLC. In addition, the anti-cancer effects of serum lipids and lipoproteins remain controversial and the mechanism is unclear [8]. Herein, the study was designed to retrospectively evaluate the prognosis significance of preoperative serum lipids and lipoproteins for NSCLC.

## Methods

Ethics Committee belonging to Liyuan Hospital affiliated to Tongji Medical College of Huazhong University of Science and Technology ratified this current retrospective study.

### Study population

Clinical and laboratory data were collected from 674 NSCLC patients (stages I-IIIA), 123 of whom were excluded from the study. The inclusion criteria of the present study were: all patients were aged ≥18 years, had pathologically diagnosed NSCLC, clinical and laboratory data were complete, and serum samples were collected before treatment. Patients were excluded when they met any of the following criteria: stage IIIB to IV or inoperable IIIA with NSCLC; use of drugs affecting lipid metabolism before serum collection; tumor of any origin other than lung; any disease associated with elevated blood lipids (diabetes, hyperlipidemia or metabolic syndrome); or blood transfusion within 4 months prior to admission. Finally, the study population comprised 551 patients (stages I-IIIA) with NSCLC confirmed by histopathology and treated from March 2013 to September 2018 in the Cancer Center at Union Hospital of Tongji Medical College of Huazhong University of Science and Technology. The pathological diagnosis and cancer stage (I, II, IIIA) were determined as per the eighth edition of TNM staging system (UICC/AJCC-8, 2017). All the histological diagnosis was determined according to the classification criteria of the WHO and the IASLC. The treatment of the patients was adherent to the national guideline (NCCN guideline Chinese version). All of 551 patients with stage I-IIIA NSCLC received curative resection, 299 of whom also underwent postoperative adjuvant chemotherapy. All subjects did not receive neoadjuvant chemotherapy, radiotherapy, and targeted therapy.

### Clinical parameters and laboratory results

Seventeen clinical parameters and laboratory results were obtained from the electronic medical records database of all subjects who were included in this study, including age, gender, smoking history, stage, histological type, surgery type, adjuvant chemotherapy, blood glucose and blood lipids examination. In strict accordance with the principle of perioperative management, all patients were required to stop smoking and drinking alcohol, and to eat a light diet, within the 2 weeks before receiving surgery. In addition, patients began fasting 8–12 h before surgery and banned drinking water 4 h before the operation. Fasting blood specimens were collected before breakfast within 7 days before surgery. All blood samples were detected using an automatic biochemical analyzer 7600 (Hitachi High-tech, Tokyo, Japan). The test items included the following: glucose, ApoB, HDL-C, ApoA1, Lpa, TC, TG, and LDL-C.

### Follow-up

All recruited patients were subjected to follow-up as per the eighth lung cancer criteria (UICC/AJCC-8, 2017). NSCLC patients discharged after treatment were subjected to follow-up, which was implemented at 3 months interval (for the initial 2 years), and subsequently at 6 months interval (for the next 3–5 years), until death or 1 December 2020. The obtained median follow-up duration was 42 months (a range of 4–91 months). OS denotes the interval from operation date to the date of death, or the final follow-up. DFS denotes the interval from the operation date to the date of disease recurrence/metastasis or death. Both of OS and DFS were obtained mainly through hospital records or telephone surveys.

### Statistical analysis

IBM SPSS statistical software 23.0 was employed to perform all statistical analysis in the study. Kolmogorov-Smirnov test was utilized to evaluate whether each continuous variable conformed to a normal distribution. Non-normally distributed continuous variables were reported as median (first-third interquartile range [IQR]). Categorical variables were shown as the percentage and analyzed by means of chi-squared test. ROC curve was calculated to determine the best cut-off value and the AUC for survival outcomes. Kaplan-Meier alongside Cox proportional hazards model were adopted for survival analysis and confirm the independent prognostic factors for NSCLC. *P* < 0.05 was suggestive of statistically significant.

## Results

### Subjects’ clinical characteristics

Table 1 depicts patients’ clinical characteristics. Enrolled in this study were 551 patients with NSCLC, including 383 (69.5%) men and 168 (30.5%) women. On the basis of the TNM criteria of the UICC/AJCC-8, the number of NSCLC patients at the stages of I-II and IIIA were, respectively, 354 (64.2%) and 197 (35.8%). All NSCLC patients received curative resection, and the number of patients received lobectomy, pneumonectomy and other were, respectively, 395 (71.7%), 120 (21.8%) and 36 (6.5%). Moreover, a total of 299 (54.3%) patients underwent postoperative adjuvant chemotherapy.

**Table 1:** The clinical characteristics of all patients (*n* = 551)

Characteristics	Patients
**Age(y)**	61 (53–68)
**Gender**	
Male	383 (69.5%)
Female	168 (30.5%)
**Smoking history**	
Y	355 (64.4%)
N	196 (35.6%)
**Stage**	
I-II	354 (64.2%)
IIIA	197 (35.8%)
**Pathological tumor**	
**classification (pT)**	
pT1–2	475 (86.2%)
pT3–4	76 (13.8%)
**Pathological lymph node**	
**stage (pN)**	
pN0	298 (54.1%)
pN1–2	253 (45.9%)
**Histological type**	
Squamous cell carcinoma	174 (31.6%)
Adenocarcinoma	329 (59.7%)
Large cell carcinoma	48 (8.7%)
**Surgery type**	
Lobectomy	395 (71.7%)
Pneumonectomy	120 (21.8%)
Other	36 (6.5%)
**Adjuvant chemotherapy**	
Y	299 (54.3%)
N	252 (45.7%)
**GLU (mmol/L)**	5.27 (4.78–5.88)
**TC (mmol/L)**	4.20 (3.63–4.89)
**TG (mmol/L)**	1.09 (0.80–1.72)
**HDL-C (mmol/L)**	1.17 (0.97–1.40)
**LDL-C (mmol/L)**	2.55 (2.08–3.08)
**ApoA1(g/L)**	1.15 (1.01–1.29)
**Apo B(g/L)**	0.85 (0.71–1.02)
**Lpa (mg/L)**	189 (90–408)

Continuous variables were shown as median (first-third interquartile range [IQR])

Categorical variables were shown as percentages

### Optimal cut-off value for preoperative lipid parameters

**Fig. 1:**
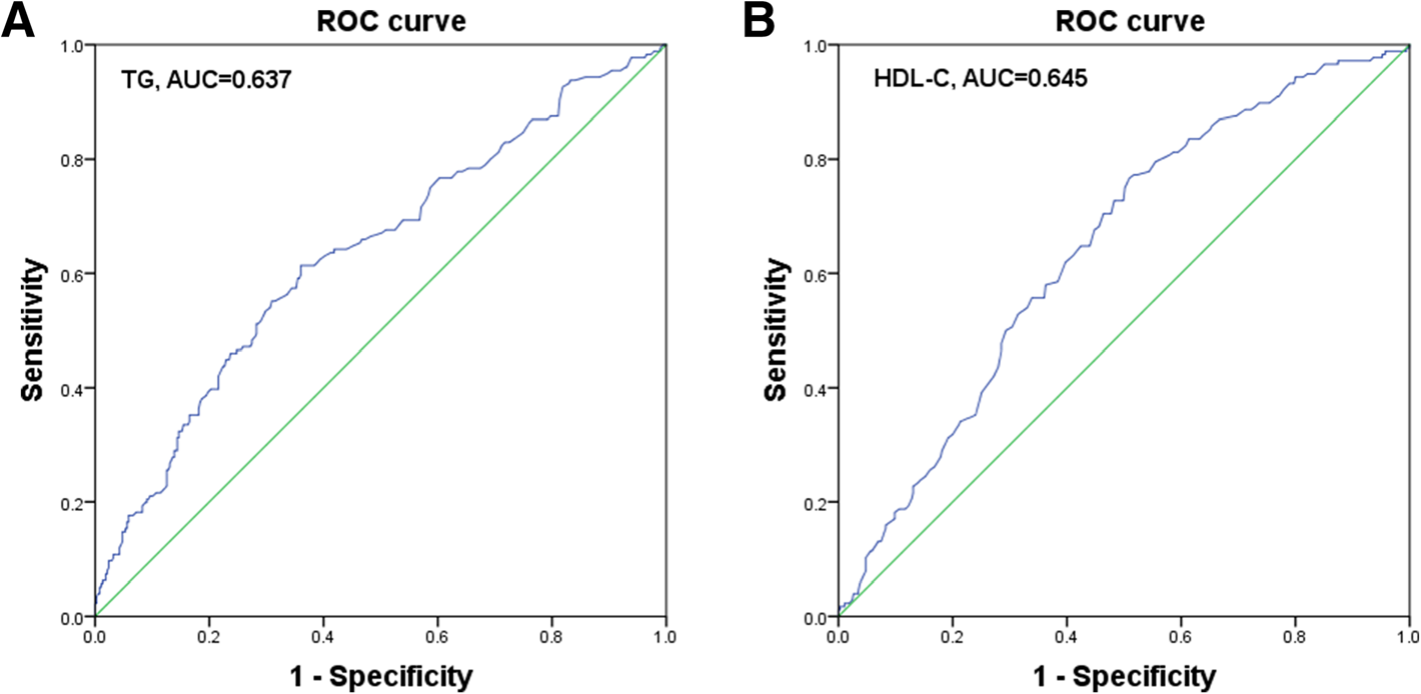
ROC curve analysis of preoperative (**A**) TG and (**B**) HDL-C for predicting survival outcomes in patients with NSCLC

To determine the optimal cut-off value and the AUC for preoperative lipid parameters in NSCLC patients, ROC curve was plotted for survival outcomes. The cut-off value of joint maximum sensitivity and specificity for TG was 1.21 mmol/L (*P* < 0.001, AUC = 0.637, 95% CI = 0.586–0.687; Fig. 1A) and HDL-C was 1.26 mmol/L (*P* < 0.001, AUC = 0.645, 95% CI = 0.597–0.692; Fig. 1B). For further analyses, the patients were assigned into high TG, or low TG group (> 1.21 mmol/L or ≤ 1.21 mmol/L, respectively), and high HDL-C, or low HDL-C group (> 1.26 mmol/L or ≤ 1.26 mmol/L, respectively).

### Clinical data analysis based on TG and HDL-C

As shown in Table 2, the clinical data of patients disaggregated by the different levels of preoperative TG and HDL-C were evaluated by the chi-squared test. The results presented that TG levels were correlated with stage (*P* = 0.018), pN (*P* = 0.001) and adjuvant chemotherapy (*P* = 0.002). However, there appeared no statistical differences between the high TG and low TG groups in age, gender, smoking history, pT, histological type, surgery type (*P* > 0.05 for all). HDL-C level linked to the gender (*P* = 0.010) and stage (*P* = 0.046).

**Table 2:** Relationships between clinicopathological data and the different levels of preoperative TG and HDL-C in 551 NSCLC patients

Characteristics	TG ≤ 1.21 mmol/L	TG>1.21 mmol/L	***P*** value	HDL-C ≤ 1.26 mmol/L	HDL-C>1.26 mmol/L	***P*** value
	***n*** = 311	***n*** = 240		***n*** = 330	***n*** = 221	
**Age**			0.842			0.112
≤ 57	114	86		111	89	
>57	197	154		219	132	
**Gender**			0.100			0.010
Male	225	158		243	140	
Female	86	82		87	81	
**Smoking history**			0.670			0.944
Y	198	157		213	142	
N	113	83		117	79	
**Stage**			0.018			0.046
I-II	213	141		201	153	
IIIA	98	99		129	68	
**Pathological tumor**						
**Classification (pT)**			0.823			0.380
pT1–2	269	206		281	194	
pT3–4	42	34		49	27	
**Pathological lymph node**						
**stage (pN)**			0.001			0.068
pN0	188	110		168	130	
pN1–2	123	130		162	91	
**Histological type**			0.522			0.121
Squamous cell carcinoma	101	73		109	65	
Adenocarcinoma	180	149		187	142	
Large cell carcinoma	30	18		34	14	
**Surgery type**			0.082			0.174
Lobectomy	233	162		227	168	
Pneumonectomy	57	63		80	40	
Other	21	15		23	13	
**Adjuvant chemotherapy**			0.002			0.610
Y	151	148		182	117	
N	160	92		148	104	

Data were present with Chi-square test. *P* < 0.05 was considered significant

### Prognostic significance of preoperative TG and HDL-C

**Fig. 2:**
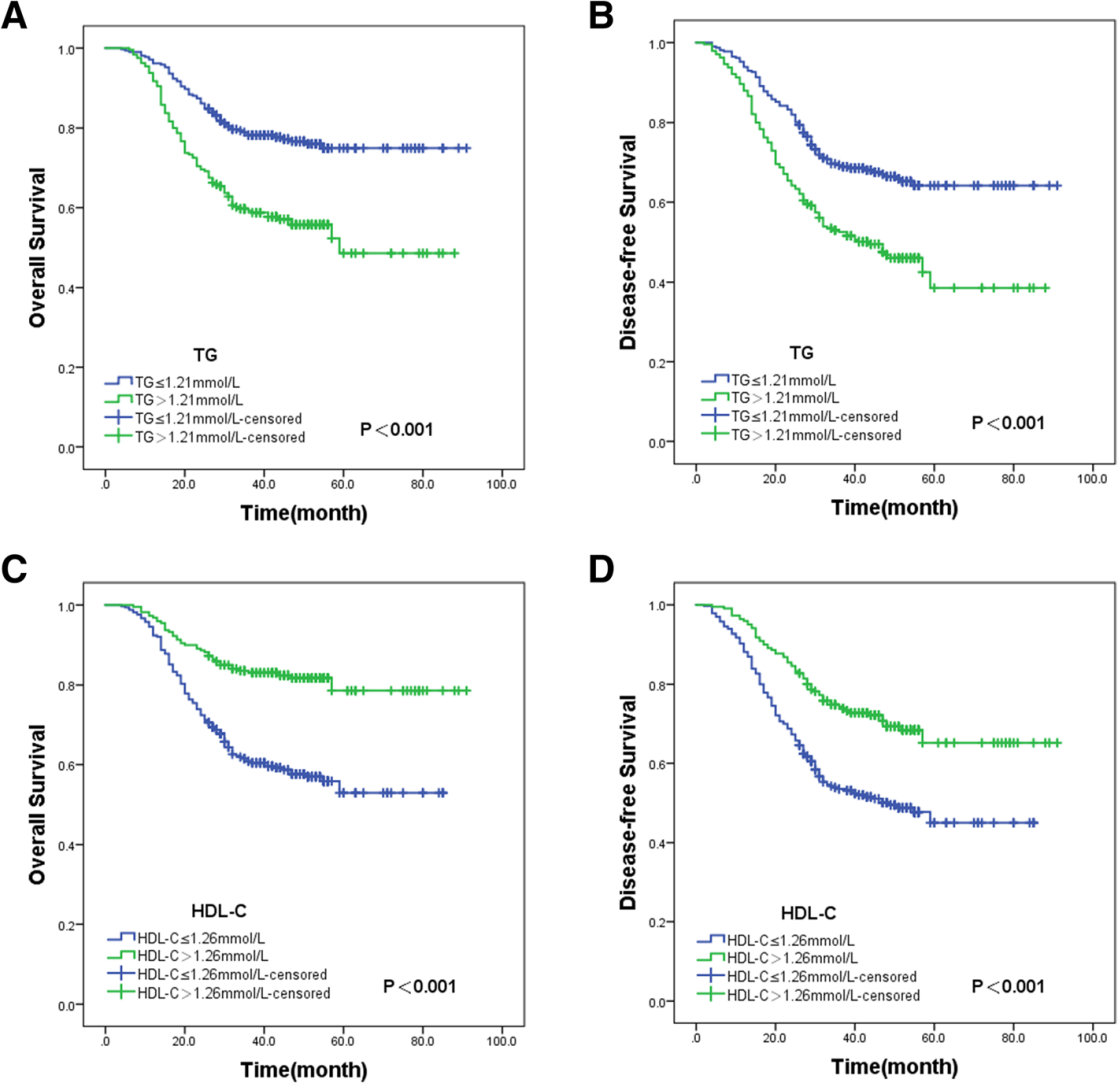
Kaplan-Meier curve of the (**A**) OS along with (**B**) DFS as per preoperative TG levels, and for (**C**) OS and (**D**) DFS as per preoperative HDL-C levels

Kaplan-Meier curve was plotted to perform the survival analysis. In relation to patients assigned in the low TG group, those in the high TG group had shorter OS (*P* < 0.001; Fig. 2A) alongside decreased DFS (*P* < 0.001; Fig. 2B). Meanwhile, the low HDL-C group had shorter OS (*P* < 0.001; Fig. 2C) alongside decreased DFS (*P* < 0.001; Fig. 2D) relative to the high HDL-C group.

Analysis on the OS (Table 3) and DFS (Table 4) was further carried out by univariate and multivariate Cox proportional hazards models. Univariate Cox proportional hazards model suggested association of age, stage, pT, pN, surgery type, adjuvant chemotherapy, HDL-C, TG, and LDL-C with OS (*P* < 0.05 for all). The multivariate model unveiled that age (*P* = 0.013), pN (*P* < 0.001), adjuvant chemotherapy (*P* = 0.002), TG (*P* < 0.001), and HDL-C (*P* < 0.001) worked as prognosis factors for OS in an independent fashion. For DFS, the univariate model suggested association of gender, stage, pT, pN, surgery type, adjuvant chemotherapy, HDL-C, TG, and LDL-C with DFS (*P* < 0.05 for all). In addition, the multivariate model uncovered gender (*P* = 0.008), stage (*P* = 0.001), pN (*P* < 0.001), adjuvant chemotherapy (*P* < 0.001), TG (*P* = 0.002), and HDL-C (*P* < 0.001) as prognosis factors for DFS in an independent fashion.

**Table 3:** Univariate and multivariate Cox proportional hazards model analysis for overall survival

Characteristics	Univariate analysis			Multivariate analysis		
	HR	95%CI	***P*** value	HR	95%CI	***P*** value
**Age**			0.023			0.013
≤ 57	1.000	Reference		1.000	Reference	
>57	1.458	1.053–2.020		1.524	1.091–2.129	
**Gender**			0.139			
Male	1.000	Reference				
Female	0.777	0.556–1.086				
**Smoking history**			0.120			
Y	1.000	Reference				
N	0.775	0.563–1.068				
**Stage**			<0.001			0.051
I-II	1.000	Reference		1.000	Reference	
IIIA	3.075	2.279–4.148		1.747	0.997–3.060	
**Pathological tumor**						
**Classification (pT)**			0.009			0.285
pT1–2	1.000	Reference		1.000	Reference	
pT3–4	1.663	1.138–2.430		1.295	0.806–2.078	
**Pathological lymph node**						
**stage (pN)**			<0.001			<0.001
pN0	1.000	Reference		1.000	Reference	
pN1–2	3.842	2.772–5.323		4.574	2.694–7.767	
**Histological type**			0.136			
Squamous cell carcinoma	1.000	Reference				
Adenocarcinoma	0.847	0.611–1.173				
Large cell carcinoma	1.371	0.815–2.305				
**Surgery type**			<0.001			0.613
Lobectomy	1.000	Reference		1.000	Reference	
Pneumonectomy	2.379	1.719–3.293		1.250	0.760–2.056	
Other	2.231	1.316–3.781		1.026	0.543–1.938	
**Adjuvant chemotherapy**			<0.001			0.002
Y	1.000	Reference		1.000	Reference	
N	2.190	1.596–3.006		2.607	1.439–4.723	
**GLU (mmol/L)**			0.115			
≤ 5.75	1.000	Reference				
>5.75	0.761	0.542–1.068				
**TC (mmol/L)**			0.490			
≤ 4.59	1.000	Reference				
>4.59	0.896	0.655–1.225				
**TG (mmol/L)**			<0.001			<0.001
≤ 1.21	1.000	Reference		1.000	Reference	
>1.21	2.217	1.640–2.997		1.891	1.385–2.583	
**HDL-C (mmol/L)**			<0.001			<0.001
≤ 1.26	1.000	Reference		1.000	Reference	
>1.26	0.371	0.260–0.527		0.430	0.301–0.613	
**LDL-C (mmol/L)**			0.046			0.742
≤ 2.26	1.000	Reference		1.000	Reference	
>2.26	1.399	1.006–1.945		1.059	0.752–1.492	
**ApoA1(g/L)**			0.181			
≤ 1.21	1.000	Reference				
>1.21	0.811	0.597–1.102				
**ApoB(g/L)**			0.222			
≤ 1.15	1.000	Reference				
>1.15	0.726	0.434–1.214				
**Lpa (mg/L)**			0.374			
≤ 53	1.000	Reference				
>53	0.828	0.546–1.256				

Data were analyzed by Cox proportional hazards model. *P* < 0.05 was considered significant

**Table 4:** Univariate and multivariate Cox proportional hazards model analysis for disease-free survival

Characteristics	Univariate analysis			Multivariate analysis		
	HR	95%CI	***P*** value	HR	95%CI	***P*** value
**Age**			0.050			
≤ 57	1.000	Reference				
>57	1.323	1.000–1.751				
**Gender**			0.021			0.008
Male	1.000	Reference		1.000	Reference	
Female	0.704	0.522–0.948		0.664	0.491–0.898	
**Smoking history**			0.270			
Y	1.000	Reference				
N	0.856	0.649–1.128				
**Stage**			<0.001			0.001
I-II	1.000	Reference		1.000	Reference	
IIIA	4.100	3.132–5.367		2.367	1.433–3.909	
**Pathological tumor**						
**Classification (pT)**			0.001			0.577
pT1–2	1.000	Reference		1.000	Reference	
pT3–4	1.726	1.234–2.415		1.124	0.746–1.694	
**Pathological lymph node**						
**stage (pN)**			<0.001			<0.001
pN0	1.000	Reference		1.000	Reference	
pN1–2	4.507	3.368–6.032		5.174	3.122–8.575	
**Histological type**			0.248			
Squamous cell carcinoma	1.000	Reference				
Adenocarcinoma	0.902	0.677–1.203				
Large cell carcinoma	1.315	0.815–2.122				
**Surgery type**			<0.001			0.082
Lobectomy	1.000	Reference		1.000	Reference	
Pneumonectomy	2.874	2.166–3.814		1.392	0.915–2.118	
Other	2.173	1.341–3.519		0.827	0.472–1.449	
**Adjuvant chemotherapy**			<0.001			<0.001
Y	1.000	Reference		1.000	Reference	
N	2.605	1.960–3.462		3.050	1.723–5.397	
**GLU (mmol/L)**			0.357			
≤ 5.75	1.000	Reference				
>5.75	0.873	0.654–1.166				
**TC (mmol/L)**			0.779			
≤ 4.59	1.000	Reference				
>4.59	0.962	0.733–1.263				
**TG (mmol/L)**			<0.001			0.002
≤ 1.21	1.000	Reference		1.000	Reference	
>1.21	1.876	1.444–2.436		1.544	1.177–2.025	
**HDL-C (mmol/L)**			<0.001			<0.001
≤ 1.26	1.000	Reference		1.000	Reference	
>1.26	0.496	0.372–0.661		0.593	0.444–0.793	
**LDL-C (mmol/L)**			0.027			0.565
≤ 2.26	1.000	Reference		1.000	Reference	
>2.26	1.384	1.038–1.845		1.091	0.810–1.470	
**ApoA1(g/L)**			0.390			
≤ 1.21	1.000	Reference				
>1.21	0.890	0.682–1.161				
**ApoB(g/L)**			0.126			
≤ 1.15	1.000	Reference				
>1.15	0.704	0.449–1.103				
**Lpa (mg/L)**			0.344			
≤ 53	1.000	Reference				
>53	0.839	0.582–1.207				

Data were analyzed by Cox proportional hazards model. *P* < 0.05 was considered significant

## Discussion

The interaction between lipid imbalance and cancer microenvironment is complex [20]. Abnormal lipid metabolism is related to the formation and development of tumor and possible to become a new target of anti-cancer [21]. Many studies have suggested that lipid molecules and their derivatives are associated with the morbidity and mortality of some cancers, such as the cancer of breast, stomach, colorectal and prostate [7, 15]. However, few studies have investigated the significance of dyslipidemia and lipoprotein metabolism in the prognosis of NSCLC.

In the present analysis, we found that preoperative serum TG and HDL-C can work as independent prognosis factors for DFS and OS in patients succumbed to NSCLC. Besides, patients with relatively high preoperative TG levels (> 1.21 mmol/L) and low preoperative HDL-C levels (≤ 1.26 mmol/L) were associated with shorter OS and DFS. A significant association was confirmed between the decreased preoperative serum TG levels and augmented preoperative serum HDL-C level and the favorable prognosis in patients succumbed to NSCLC.

Of note, the current study first suggested that relatively high preoperative TG levels correlated to poor outcomes in patients with stages I-IIIA NSCLC. Moreover, the present findings are consistent with some previous studies. A meta-analysis of Lin et al. [22] showed even lower serum HDL-C levels in patients succumbed to lung cancer than that of healthy controls, but serum TG levels were much higher. Additionally, some previous studies have indicated that abnormalities in serum lipids and lipoproteins, such as preoperative low levels of TC, HDL-C, ApoA1 or high TG levels, indicated poor prognosis in cancer patients [7, 16, 23, 24]. This study proved that increased preoperative serum HDL-C level could work as a favorable prognostic factor for NSCLC patients, which was consistent with the study of Chi et al. [25] in NSCLC. Furthermore, Khurana et al. [26] reported that statins with anti-lipidemic effects used more than 6 months are a cause of a 55% reduction in the lung cancer risk. This further indicates that a correlation between serum lipids and lung cancer.

The present study suggested that TG and HDL-C are effective pre-treatment biomarkers for predicting prognosis of NSCLC. However, as new indicators for evaluating OS and DFS, the potential mechanism of preoperative serum TG and HDL-C to reflect the survival outcomes of NSCLC is unclear. Only a preliminary interpretation can be given through considering previous studies. Observations to date have shown that oxidative stress and inflammation are closely related to cancer [27]. The mechanism of TG in cancer is that high levels of serum TG can upregulate cell signal pathways (such as MEK/ERK and AKT pathways), which promote the development of oxidative stress, and reactive oxygen species, thereby leading to the proliferation and progression of cancer cells [12, 28, 29]. Zuber et al. [30] found that one genetic locus at 6p22.1 (rs6904596, ZNF184) in humans was associated with both NSCLC and TG. This suggested that genes that affect lipid metabolism may also affect the development and outcome of cancer.

Moreover, abnormal serum cholesterol levels cause immune system disorders, protein dysfunction, and redox disorders [17]. These factors may increase tumor angiogenesis and tumor cell proliferation, and reduce tumor cell apoptosis [31, 32]. HDL-C can abolish cholesterol transport and has anti-inflammatory, antioxidant, and anti-proliferative effects [33]. Therefore, weak serum HDL-C levels may weaken its protective property in cancer initiation, and the disordered cholesterol metabolism by the decreased HDL-C levels may be a cause of cancer progression [9, 34–37]. Besides, tissue growth, including tumor tissue, uses extra cholesterol to support membrane biosynthesis [31]. During the rapid growth of a tumor, the decreased HDL-C levels in the tumor may be due to the increased demand for cholesterol by tumor cells and the decrease of cholesterol efflux [25, 38]. In addition, under a condition of high TG levels, the net movement of TG to HDL-C molecules could increase, and triglycerides-enriched HDL-cholesterol was more easily cleared from the blood, resulting in a decrease in serum HDL-C levels [39]. The low levels of HDL-C not only weakened anti-inflammatory and antioxidant effects, but also reduced the effect of EGFR-TKI treatment, thereby leading to poorer survival outcomes [40, 41]. Collectively, the mechanisms described above seem to partly explain why preoperative serum HDL-C and TG may be the potential indicator to predict prognosis for NSCLC. Although the prognostic values of TG and HDL-C serum levels in NSCLC have been reported previously, the used detection methods, sample size, follow-up and comparison objects for analysis showed great difference. This study represents the first evidence to reveal that high TG level or weak HDL-C levels increased the risk of NSCLC significantly.

### Strength and limitations

The current study demonstrated that abnormalities in the serum lipoproteins and lipids were directly associated with the survival outcome of NSCLC patients and the level of preoperative serum HDL-C and TG can be promising biomarkers for the better prognosis of NSCLC patients.

Certainly, some limitations also exist in this study. First, as a retrospective design, this research is vulnerable to bias in data selection and analysis. Second, the sample size is relatively small, especially concerning the patients at stage IIIA or with large-cell lung cancer histological subtypes and the data may not be representative of all patients at these status. Last, the obtained findings still need large-scale prospective studies and clinical trials to verify.

## Conclusion

In summary, this study shows that lipid and lipoprotein metabolism abnormalities are associated with the survival outcomes of NSCLC. Preoperative serum HDL-C and TG may be promising biomarkers to predict OS and DFS of patients with NSCLC. Besides, patients with relatively high preoperative TG levels and relatively low preoperative HDL-C levels have poorer OS and DFS. The decreased preoperative TG levels and increased preoperative HDL-C levels were significantly linked to favorable prognosis in patients succumbed to NSCLC. Therefore, serum HDL-C and TG levels are potential clinical prognosis factor for patients succumbed to NSCLC. Furthermore, these findings might assist clinicians in recognizing the patients with the increase risk of NSCLC and establishing a framework for future individualized therapy for patients with NCSLC.

## Data Availability

Not applicable.

## Abbreviations

NSCLC: Non-small cell lung cancer
GLU: Glucose
TG: Triglycerides
LDL-C: Low-density lipoprotein cholesterol
TC: Total cholesterol
TG: Triglycerides
HDL-C: High-density lipoprotein cholesterol
LDL-C: Low-density lipoprotein cholesterol
ApoA1: Apolipoprotein A-1
ApoB: Apolipoprotein B
Lpa: Lipoprotein(a)
TNM: Tumor-node-metastasis
UICC: Union for international cancer control
WHO: World health organization
AJCC: American joint committee on cancer
WHO: World health organization
IASLC: International association for the study of lung cancer
NCCN: National comprehensive cancer network
OS: Overall survival
DFS: Disease-free survival
ROC: Receiver operative characteristic
AUC: Area under curve
HR: Hazard ratio
CI: Confidence interval
